# Correlation Between Ultrasound and Cytological Findings of Patients With Suspicious Thyroid Nodules: The King Hamad University Hospital Experience

**DOI:** 10.7759/cureus.22877

**Published:** 2022-03-05

**Authors:** Raneem Alshaikh, Khalid Almaghribi, Dhaidan M Alshammari, Hosameldin Mohamad, Wael Ebrahim, Shuruq M Alshammari, Omar Sabra

**Affiliations:** 1 Otolaryngology - Head and Neck Surgery, King Hamad University Hospital, Muharraq, BHR; 2 Radiology, King Hamad University Hospital, Muharraq, BHR; 3 ENT, Ministry of Health, Dammam, SAU; 4 Research Department, Northern Border University, Arar, SAU

**Keywords:** benign nodules, tirads scoring, bethesda system, fine needle aspiration, thyroid nodules

## Abstract

Background

Thyroid nodules are a common presentation in otolaryngology-head and neck clinics. The detection of thyroid nodules has increased significantly with the advancements in radiological technology such as computed tomography and ultrasound (US). The present study aims to improve the clinical practice and management of thyroid disorders by establishing correlations between US and cytological findings in the diagnosis of thyroid nodules.

Methodology

A retrospective cohort study was conducted at the King Hamad University Hospital (KHUH), Bahrain. A total of 189 cases met the study criteria. Pathological records for thyroid nodule fine needle aspiration (FNA) cytology and US features of sampled nodules from the patients were obtained. The cytological results were categorized into the Bethesda grading system, while the US features were organized into internationally accepted features using the Thyroid Imaging Reporting and Data System (TIRADS).

Results

The radiologic characteristics from US showed positive features largely for the composition (76.2%) and vascularity (59.3%). Very few showed echogenicity (6.9%). Most records indicated negatively for the shape (94.7%), margins (76.2%), echogenicity (63.5%), or echogenic foci (66.1%). Of the 47 cases in TIRADS 1 and 2, only two were found to be Bethesda 4 classification, showing that most of these nodules were benign. Among those with TIRADS 3 on US, 85% turned were benign (Bethesda 2), two of the remaining six were grade 3, and the other four were suspiciously malignant. Of the 100 cases in TIRADS 4 and 5, 63% were of Bethesda grade 2, and therefore, benign, 14% were mildly suspicious, and only 23% were in Bethesda grades 4-6. A significant positive correlation was noted between the TIRADS and Bethesda scores (r = 0.338, p ≤ 0.001).

Conclusions

If the thyroid nodules are classified properly by US using the TIRADS system, the probability of a nodule being malignant can be established with a certain level of confidence. The appropriate management of the nodule can be initiated avoiding unwarranted FNA procedures.

## Introduction

Nodules in the thyroid are common with a global prevalence of 4-8%. The majority of the nodules are benign, with the risk of malignancy being 7-15% [1]. Characterized by uncontrolled growth of an area within normal thyroid tissue with structural and/or functional transformation, nodular thyroid diseases are categorized as complex diseases. Thyroid nodules can be a simple nodule in the absence of associated thyroid ailments such as dysfunction, autoimmune disease, or malignancy [2,3]. Although essential, the clinical evaluation of the size of the nodule, its morphology, and function remains highly imprecise, wherein up to 50% of patients with a solitary palpable thyroid nodule are seen to have multiple nodules on ultrasound (US). Moreover, on regular palpation, up to 50% of patients can have a normal gland but have thyroid nodules when examined using US. This explains the increasing use of imaging in patients with suspicious thyroid-associated symptoms and signs [4,5].

The detection of thyroid nodules has increased significantly with the widespread use of various imaging modalities, such as computed tomography (CT), magnetic resonance imaging (MRI), and US [6]. With imaging techniques becoming more sensitive in finding smaller lesions, the incidence of thyroid nodules is expected to increase [7]. Current high-resolution imaging modalities have shown that nearly 70% of the population have at least one small nodule [8]. The US features of thyroid nodules do not always coincide with the cytological results, leading to discrepancies and confusion in management protocols [9,10].

There is an ongoing debate regarding the appropriate indication of when and which nodules are to be subjected to fine-needle aspiration cytology (FNAC). The past two decades have witnessed several controversies regarding the malignant characteristics that crop up, yet, there is no definitive classification [11]. The confusion surrounding the various classifications in the various modalities has led researchers to attempt to establish a correlation between the various techniques of staging thyroid nodules. In 2014, Canberk et al. [12] explored the utility of the Thyroid Imaging Reporting and Data System (TIRADS) as a supplementary method to FNAC in the management of thyroid nodules. They concluded that although significant associations were present among the various systems, standardization was not possible or practical.

This study aims to review our clinical practice and management of thyroid disorders by establishing correlations between US and cytological findings in the diagnosis of thyroid nodules at our center. Specifically, the objective was to understand how often highly suspicious radiological features of thyroid nodules (solid or mixed composition, hypoechogenicity, central vascularity, taller than wider in shape, irregular margins, and evidence of extrathyroid extension as positive US features) correlated with cytological malignant features according to the Bethesda classification system [13-15].

## Materials and methods

A retrospective cohort study was conducted at the King Hamad University Hospital (KHUH) Bahrain, after obtaining appropriate approval from the Institutional Review Board and Ethics Committee (approval number: 20-310). Data from medical records of all patients with thyroid nodules who underwent FNAC over three years in KHUH (2017-2019) were investigated. All patients who required both FNAC and US were selected. Records of any patient with FNAC or US done outside KHUH and/or who underwent previous thyroid surgery were excluded from the study.

A retrospective review was done of the data and medical records of all patients who met the inclusion and exclusion criteria. A total of 189 cases fit the study parameters. Pathological record results for thyroid nodule FNAC and US features of sampled nodules from the patients were recorded. The cytological results were categorized into the Bethesda grading system while suspicious US imaging findings were screened for (solid or mixed composition, hypoechogenicity, central vascularity, taller than wider in shape, irregular margins, and evidence of extrathyroid extension as positive US features). All collected data were entered in Microsoft Excel sheets and analyzed accordingly.

Statistical analyses were performed using SPSS version 28 (IBM Corp., Armonk, NY, USA), and data were tabulated in spreadsheets. Standard descriptive statistics such as mean and standard error were employed to summarize the continuous measures. Frequencies and percentages were used for the categorical measures. We used correlation coefficient, sig two-tailed, and odds ratio to correlate our results.

## Results

This retrospective study investigated 189 participants aged 15 to 86. The majority of the participants were females (84.7%, n = 160), and the mean age was 49.1 ± 14.9 years. Table 1 displays the sociodemographic characteristics of the studied population. Thyroid nodules were predominant in the third to sixth decade. The study population had 127 patients in the 35-64-year age group, amounting to more than three-fourths of the entire population.

**Table 1:** Sociodemographic characteristics of participants.

	n	%
Gender		
Female	160	84.70%
Male	29	15.30%
Age groups (years)		
15–24	11	5.80%
25–34	26	13.80%
35–44	30	15.90%
45–54	49	25.90%
55–64	48	25.40%
>64	25	13.20%

The radiologic characteristics (Table 2) from the US samples taken showed positive features largely for the composition (76.2%) and vascularity (59.3%). Very few showed echogenicity (6.9%). Most records indicated negatively for the shape (94.7%), margins (76.2%), echogenicity (63.5%), or echogenic foci (67.7%). The exclusions to these involve 53 participants, for whom echogenicity could not be determined, and 31 who did not have vascularity features examined. Three participants had comet tail artifacts, which is classified as neither positive nor negative indicator; however, overall, echogenic foci were negative in most patients (67.7%).

**Table 2:** Radiologic characteristics of participants. *: 53 participants could not have their echogenicity determined; **: 31 participants did not have this radiological feature measured.

Characteristic	Positive n (%)	Negative n (%)
Composition	144 (76.2%)	45 (23.8%)
Echogenicity*	13 (6.9%)	120 (63.5%)
Shape	10 (5.3%)	179 (94.7%)
Margin	45 (23.8%)	144 (76.2%)
Vascularity**	112 (59.3%)	46 (24.3%)
Echogenic foci	61 (32.3%)	128 (67.7%)

When the individual characteristics of the radiologic features such as sensitivity, specificity, positive predictive value (PPV), negative predictive value (NPV), and accuracy were examined (Table 3), it was evident that the maximum sensitivity (100%) and NPV (100%) was seen in the composition, while maximum specificity (95.9%), PPV (30%), and accuracy (88.89%) were present in the shape of the nodule. Although showing 100% sensitivity, the composition of the nodules on radiologic imaging only had 26.1% specificity and 32.8% accuracy. Echogenicity had a high specificity (89.92%), high NPV (89.15), and high accuracy (81.2%), as did echogenic foci, with all high characteristics except the PPV (18.3%). Vascularity also showed greater levels of sensitivity (88.9%) and NPV (97.8%). However, accuracy (33.5%) and specificity (30.2%) were only a third, and even lower was the PPV (7.1%).

**Table 3:** Sensitivity, specificity, PPV, NPV, and accuracy of radiological parameters: composition, echogenicity, shape, margin, vascularity, and echogenic foci. PPV: positive predictive value; NPV: negative predictive value

	Sensitivity	Specificity	PPV	NPV	Accuracy
Composition	100% (80.4–100%)	26.1% (19.7–33.4%)	11.81% (10.9–12.7%)	100% (99.8–100%)	32.8% (26.1–39.9%)
Echogenicity	7.14% (0.2–33.8%)	89.92% (83.5–94%)	7.7% (1.1–37.2%)	89.1% (87.5–90.5%)	81.2% (73.5–87.4%)
Shape	17.6% (3.8–43.4%)	95.9% (91.7–98.3%)	30.0% (10.8–60.1%)	92.1% (90.4–93.6%)	88.89% (83.5–92.9%)
Margin	58.8% (32.9–81.5%)	79.6% (72.8–85.4%)	22.2% (14.8–31.9%)	95.1% (91.6–97.2%)	77.7% (71.1–83.4%)
Vascularity	88.9% (51.7–99.7%)	30.2% (22.9–38.2%)	7.1% (5.6–9.0%)	97.8% (87.4–99.6%)	33.5% (26.2–41.4%)
Echogenic foci	64.7% (38.3–85.7%)	70.4% (62.9–77.2%)	18.3% (12.6–25.1%)	95.2% (91.2–97.4%)	69.9% (62.7–76.4%)

**Figure 1:**
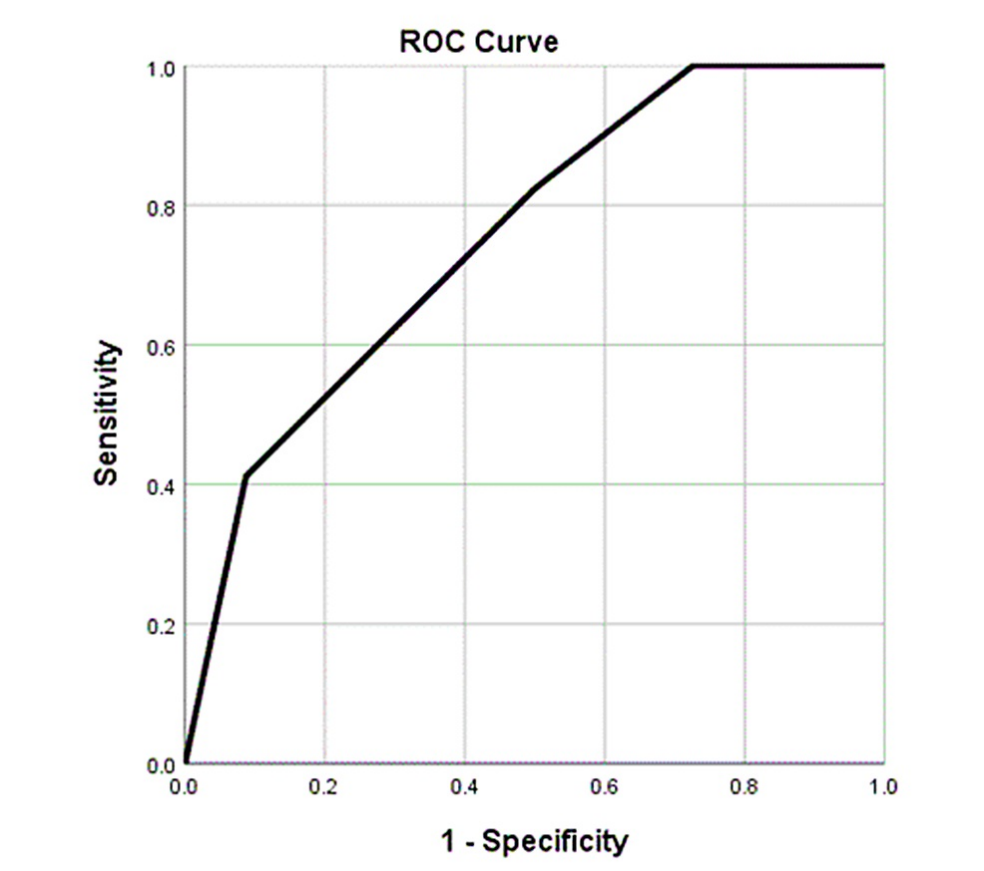
ROC curve analysis: TIRADS. ROC: receiver operating characteristic; TIRADS: Thyroid Imaging Reporting and Data System

The TIRADS receiver operating characteristic curve analysis (Figure 1) revealed 75.3% area under the curve. A cut-off value of TIRADS 3.5 showed 82.4% sensitivity and 50% specificity in identifying malignant cases.

The correlation between TIRADS and Bethesda classifications (Table 4) of the 189 participants did not include those who were already a proven malignancy (TIRADS 6). Under TIRADS 2, there were 24 cases, and 19 cases were in the TIRADS 1-2 score category. TIRADS 3-5 categories had 42, 78, and 22, cases respectively. Nodules classified under Bethesda I and II were benign, while those under Bethesda IV-VI were malignant. A total of 144 cases were in the Bethesda 2 category, and the others had an almost even distribution of cases. Only three cases were found to be of Bethesda V classification. TIRADS 1-2 were not suspicious of malignancy, leaning toward benign pathology. TIRADS 3-5 were suspicious of malignant pathology, ranging from mild to high possibility.

**Table 4:** TIRADS and Bethesda correlation. TIRADS: Thyroid Imaging Reporting and Data System

	TIRADS n (%)						Total
Bethesda	1	1–2	2	3	4	5	
2	4 (2.8%)	19 (13.2%)	22 (15.3%)	36 (25.0%)	53 (36.8%)	10 (6.9%)	144
3	0 (0%)	0 (0%)	0 (0%)	2 (12.5%)	12 (75.0%)	2 (12.5%)	16
4	0 (0%)	0 (0%)	2 (16.7%)	1 (8.3%)	6 (50.0%)	3 (25.0%)	12
5	0 (0%)	0 (0%)	0 (0%)	0 (0%)	2 (66.7%)	1 (33.3%)	3
6	0 (0%)	0 (0%)	0 (0%)	3 (21.4%)	5 (35.7%)	6 (42.9%)	14
	4 (2.1%)	19 (10.1%)	24 (12.7%)	42 (22.2%)	78 (41.3%)	22 (11.6%)	189

Of the 47 cases in TIRADS 1-2, only two cases were found to be Bethesda 4 classification, showing that most of these nodules turned out to be benign. Among those with TIRADS 3 on US, 85% were benign (Bethesda 2), two of the remaining six were of grade 3, and the other four were suspiciously malignant. Of the 100 cases in TIRADS 4 and 5, 63% were of Bethesda grade 2, and thereby benign, 14% mildly suspicious, and only 23% were in the Bethesda grades 4-6.

Considering the above, the correlation of TIRADS characteristics with Bethesda 5 and 6 (Table 5) shows significant weak positive correlation was noted with composition (r = 0.176, p = 0.016) and shape (r = 0.174, p = 0.017). Comparatively, margin and echogenic foci had a slightly greater positive correlation to Bethesda 5 and 6 grades. A significant positive correlation was noted between the TIRADS and Bethesda scores (r = 0.338, p < 0.000).

**Table 5:** Correlation of TIRADS characteristics with Bethesda grades 5 and 6. TIRADS: Thyroid Imaging Reporting and Data System

	Composition	Echogenicity	Shape	Margin	Echogenic foci
Correlation coefficient	0.176	-0.30	0.174	0.258	0.211
Sig. (two-tailed)	0.016	0.728	0.017	0.000	0.004

Judging the risk of malignancy from the correlations established (Table 6), it was evident that for individuals with TIRADS 4 classification, the risk of malignancy could be estimated to be one or two-fold compared to that for patients rated classification 3 (95% confidence interval (CI:) -0.3-5.2); however, probably owing to the small sample size this finding was not significant. For those having a TIRADS score of 5, however, the risk of malignancy was estimated at 6.06-fold the risk for those rated as 3 (95% CI: 1.3-26.6).

**Table 6:** TIRADS and the correlation with the risk of malignancy. TIRADS: Thyroid Imaging Reporting and Data System

TIRADS classification	Benign	Malignant	Total	OR (95% CI)	P-value
	n (%)	n (%)	n		
1	4 (100.0%)	0 (0.0%)	4	0.04 (0.001-0.96)	0.04
1, 2	19 (100.0%)	0 (0.0%)	19	0.17 (0.01-3.7)	0.26
2	24 (100.0%)	0 (0.0%)	24	0.22 (0.01-4.7)	0.33
3	39 (92.9%)	3 (7.1%)	42	Reference	
4	71 (91.0%)	7 (9.0%)	78	1.2 (0.3-5.2)	0.72
5	15 (68.2%)	7 (31.8%)	22	6.06 (1.3-26.6)	0.01
Total	172 (91.0)	17 (8.9)	189		

## Discussion

Evaluation of thyroid nodules must follow a quadruple assessment. A four-pronged approach begins with history taking and clinical examination, thyroid function tests, US, and cytology. The availability of several staging systems allows for more precise diagnosis and early management of malignant tissue. The endorsement of the TIRADS system makes it a unique assessment of choice for the staging of thyroid nodules. However, its correlation with the Bethesda system is ambiguous. Overlapping misses have been noted both with the TIRADS staging and the Bethesda classification [16,17]. Therefore, it is important to have some degree of forethought to cross-check in cases where malignancy is suspected.

Cytologically indeterminate thyroid nodules pose a challenge in clinical decision-making. In 2017, Delfim et al. [18] proposed a new TIRADS classification system based on their analyses of US features of benign and malignant thyroid nodules and the likelihood of malignancy associated with each feature following the Bethesda System for Reporting Thyroid Cytopathology and Histopathology. The new system proposed a cut-off score of 2 (sensitivity 97.4% and specificity 51.6%), adopted as the transition between likely benign (TR 3) and TR 4a nodules. Overall, the frequency of malignancy in thyroid nodules in compliance with the categories was 1.0% for TR3, 7.8% for TR 4a, 35.3% for TR 4b, and 84.7% for TR 5. Poller (2017) [19] concluded that the cytopathologist review of thyroid US is immensely useful, especially for patient triage in cases of solid, mixed cystic, and/or solid and pure cystic thyroid lesions with non-diagnostic or unsatisfactory thyroid FNA.

In this study, we shared our experience of using TIRADS to evaluate the risk of malignancy in thyroid nodules. Given the abovementioned results, TIRADS was a good method to rule out the presence of malignancies (Table 5). This might be considered to minimize the need for FNAC for TIRADS (1-3) in the future.

Comparable with the present findings, a study by Rahal et al. (2016) [20] showed that most nodules with a TIRADS score of 2 or 3 were mostly Bethesda grade 2 (95.5% and 92.5%, respectively). However, in this study, contrarily, the majority of the nodules with TIRADS 4 or 5 were also Bethesda 2 or 3. Moreover, two cases of unexpected malignancy in the nodules had been classified as TIRADS 2 but were found to be Bethesda 4 [21,22].

A recent observational study by George et al. (2021) found the overall concordance of US, TIRADS, Bethesda system, and histopathology to be 69.8% [23]. The malignancy rate of TR 5 nodules was found to be 97.1% with a significant p-value (0.022). This study found a six-fold risk of malignancy in TR 5 nodules compared to those with TR 3, with a p-value of 0.01. Rahal et al. reported the risk of malignancy for patients with TR 4A and 4B to be approximately 10.86-fold and 43.27-fold of those with TR 3 staging.

The majority of contemporary literature examines the diagnostic value of TIRADS with thyroid nodule cytopathological classification (Bethesda). Similar to our findings, a retrospective study by Sahli et al. [24] had around 100 cases in TR 4 or 5 categories and 25 in the TR3 categories but had a lower sensitivity (71.4%), specificity (38.1%), and NPV (69.6%) for the diagnosis of malignancy. Contrary to this, a prospective study involving 114 patients showed a high NPV (92-100%) for TR 4 and 5 categories allowing for better exclusion of malignancy in the diagnostic workup of thyroid nodules [25]. In addition, a retrospective study by Singaporewalla et al. [26] involving 100 consecutive cases compared ultrasonographic TIRADS findings to all the cytological Bethesda categories, finding a concordance rate of 83% with sensitivity (70.6%), specificity (90.4%), and NPV (93.8%).

Because diagnostic modalities of imaging play a central role in the management of thyroid nodules, the radiologist must be familiar if not well-versed with the used diagnostic categories for cytopathology and ultrasonography. The Bethesda framework and the TIRADS classification were established to provide a rational approach to the diagnosis and treatment protocols of thyroid nodules. The objective of such studies remains to correlate US features to cytological findings, with a view to increasingly graduate the risk of a nodule being malignant based on the number of features present in the US [27,28].

Because this is a retrospective study, the study parameters could not be controlled by the investigators. Moreover, owing to the small sample size and single-center study design, the findings cannot be generalized with complete assurance at the population level. Convenience sampling offers bias that cannot be excluded. These can be averted by a prospective study on a larger scale to arrive at more concrete conclusions. Another limitation of our study is that the final pathology of thyroidectomy specimens was not available which can give a more accurate diagnosis than FNAC.

## Conclusions

If the thyroid nodules are classified properly by US using the TIRADS system, the probability of a nodule being malignant can be established with a certain level of confidence. The appropriate management of nodules can be initiated avoiding unwarranted FNA procedures. The major outcome of our study is the high NPV of the TIRADS score.

The variable predicting power of the TIRADS system in different studies reflects a limitation for this system in easily following it in a reproducible fashion by different radiology groups. This puts more responsibility on each group to regularly audit their data to limit the indication of unnecessary FNACs. Our data show that a low TIRADS score is reassuring and should be a strong indication against FNAC in our setting, while a high TIRADS is less predictive. These results might be the result of an overestimation of the TIRADS by our radiology team or an under reading of the Bethesda system rating on FNAC by our pathologists.
